# Evaluating the Diagnostic Accuracy of ChatGPT-4 Omni and ChatGPT-4 Turbo in Identifying Melanoma: Comparative Study

**DOI:** 10.2196/67551

**Published:** 2025-03-21

**Authors:** Samantha S. Sattler, Nitin Chetla, Matthew Chen, Tamer Rajai Hage, Joseph Chang, William Young Guo, Jeremy Hugh

**Affiliations:** 1 Department of Dermatology, Stony Brook University Hospital, Stony Brook, NY, United States; 2 School of Medicine, University of Virginia, Charlottesville, VA, United States; 3 Renaissance School of Medicine, Stony Brook University, Stony Brook, NY, United States; 4 Virginia Tech, Blacksburg, VA, 24061, United States, 1 7038948362; 5 University of Passau, Passau, Germany

**Keywords:** melanoma, skin cancer, chatGPT, chat-GPT, chatbot, dermatology, cancer, oncology, metastases, diagnostic, diagnosis, lesion, efficacy, machine learning, ML, artificial intelligence, AI, algorithm, model, analytics

## Abstract

ChatGPT is increasingly used in healthcare. Fields like dermatology and radiology could benefit from ChatGPT’s ability to help clinicians diagnose skin lesions. This study evaluates the accuracy of ChatGPT in diagnosing melanoma. Our analysis indicates that ChatGPT cannot be used reliably to diagnose melanoma, and further improvements are needed to reach this capability.

## Introduction

Artificial Intelligence (AI) is being increasingly integrated into health care [1]. Multiple AI systems exist in medicine, including large language models (LLMs), neural networks, and predictive models. While studies have demonstrated AI’s mixed precision and accuracy, the technology is poised to assist with data-driven diagnostics in dermatology [2].

There has a been rapid popularization of the LLM, ChatGPT for home-based medical inquiries [3]. Minimal research exists on ChatGPT’s accuracy in detecting melanoma. Given that patients are increasingly presenting internet-derived diagnostics during cancer consultations, it is imperative to understand the capabilities of commonly used AI engines, such as ChatGPT [4]. In this study, we compare the capabilities of two models—ChatGPT-4 Omni (GPT-4o) and ChatGPT-4 Turbo (GPT-4 Turbo)—in identifying melanoma versus “not melanoma” skin lesions. These LLMs were chosen due to their accessibility and ability to answer image-based dermatology board-style questions correctly [5].

## Methods

OpenAI was used to query GPT-4o and GPT-4 Turbo for classifying dermatoscopic images of melanoma versus “not melanoma” (ie, melanocytic nevi, basal cell carcinoma, actinic keratoses, dermatofibromas, and vascular lesions) selected from the HAM10K database, which contains >10,000 dermatoscopic images collected over 20 years from multiple populations, and verified by histopathology or confocal microscopy [6].

Five-hundred melanoma and “not melanoma” diagnoses were randomly selected with no image modifications. A standardized prompt was used: “This is an image of the step 1 examination. The multiple-choice question is as follows: Based on the image, does the patient have (A) melanoma (B) no melanoma? Only output the answer as A or B.” Incomplete responses were categorized as “not a number” and excluded.

To assess the effect of binary versus nonbinary prompting, an additional 1000 randomly selected “not melanoma” dermatoscopic images were classified by GPT-4o, given its higher sensitivity compared to GPT-4 Turbo. Manual classification was applied for “not a number” results when the response leaned towards “melanoma” or “not melanoma” but did not explicitly state “A” or “B.”

## Results

The diagnostic accuracies of GPT-4 Turbo and GPT-4o were 0.546 (95% CI 0.515‐0.577) and 0.577 (95% CI 0.547‐0.608), respectively. There was no significant difference in accuracy between the two models (*P*=.10). GPT-4 Turbo demonstrated a sensitivity of 76.3%, specificity of 32.9%, and false-positive rate of 67.1% (Table 1). GPT-4o yielded a higher sensitivity of 96.8% (*P*<.001), lower specificity of 18.4% (*P*=.09), and higher false-positive rate of 81.6% (*P*<.001).

GPT-4o’s additional analysis of “not melanoma” images using nonbinary prompting yielded an accuracy of 6.56% (95% CI 4.94%‐8.18%), correctly classifying 59 of 899 images (Table 2). Binary prompting increased GPT-4o accuracy to 25.25% (95% CI 22.55%‐27.95%), with 252 of 998 images correctly identified as “not melanoma.” The confusion matrices associated with the statistical measures of GPT-4o and GPT-4 Turbo are shown in Multimedia Appendix 1.

**Table 1.** GPT-4 Omni and GPT-4 Turbo demonstrate low accuracy and low specificity for melanoma diagnosis.

Statistical measure	Chat-GPT 4 Turbo	Chat-GPT 4 Omni
Accuracy, (95% CI)	0.546 (0.515‐0.577)	0.577 (0.547‐0.608)
Precision	0.532	0.544
Specificity, % (95% CI)	32.9 (0.288‐0.370)	18.4 (0.150‐0.218)
Sensitivity, % (95% CI)	76.3 (0.726‐0.801)	96.8 (0.952‐0.983)
F1-score	0.627	0.697
False-positive rate (%)	67.1	81.6

**Table 2.** Accuracy of ChatGPT-4o in diagnosing melanoma and “not melanoma” with binary versus nonbinary prompting.

Statistical measure	Nonbinary prompting (n=899)	Binary prompting (n=998)
Accuracy, n (%)	59 (6.56)	252 (25.25)
95% CI (%)	4.94‐8.18	22.55‐27.95
False-positive rate (%)	81.6	67.1

## Discussion

Currently, GPT engines demonstrate low accuracy for diagnosing melanoma. Higher diagnostic accuracies have been achieved using neural networks such as Moleanalyzer pro (87.7%) and ChatGPT Vision (85%); however, these studies included much smaller sample sizes of 100 and 60 images, respectively [7,8]. Our findings exhibit a higher-powered analysis of ChatGPT performance.

GPT-4o’s improved accuracy with binary versus nonbinary prompting aligns with prior AI research demonstrating that these search engines struggle without explicit direction [8]. When more intricate prompts are provided, results improve [7,8]. However, such a methodology is not generalizable to the average user. Patients using these engines to self-diagnose suspicious lesions at home are more likely to use nonbinary prompts without detailed instructions for the AI engine. Thus, our nonbinary prompting results reflect that ChatGPT would provide inaccurate outputs when used by the average patient.

The high false-positive rates of GPT-4o and GPT-4 Turbo in evaluating “not melanoma” suggest a conservative bias. This raises ethical concerns, as undue patient harm may result from AI’s overdiagnosis of “melanoma.” Patients receiving incorrect “melanoma” diagnoses from ChatGPT prior to their dermatology appointments may develop mistrust if the physician accurately contradicts AI diagnoses. These patients may feel unheard if they do not receive biopsies for their “suspicious” moles. Increased in-office counseling may be warranted to disentangle the biases AI imparts to patients.

Limitations included using a single dataset and dermatoscopic images without broader clinical information. The models were not specifically trained before querying. ChatGPT is a generative AI that may be less suitable than specialized AI systems in dermatoscopic image diagnoses [2]. Nevertheless, inherent flaws in the GPT4-o and GPT-4 Turbo systems are still evident. Therefore, patients should avoid ChatGPT diagnoses before evaluation of their suspected pigemented lesions by trained dermatologists.

## Supplementary material

10.2196/67551 Multimedia Appendix 1 Confusion matrix of ChatGPT-4 Omni performance (top) and confusion matrix of ChatGPT-4 Turbo performance (bottom).

## Abbreviations

AI: artificial intelligence
GPT-4 Turbo: ChatGPT-4 Turbo
GPT-4o: ChatGPT-4 Omni
LLM: large language model
