# Supplementary figures and images for: Evaluating the Diagnostic Accuracy of ChatGPT-4 Omni and ChatGPT-4 Turbo in Identifying Melanoma: Comparative Study
Source: JMIR Dermatol. 2025 Mar 21;8:e67551. doi: 10.2196/67551 (PMC11952272; doi:10.2196/67551)

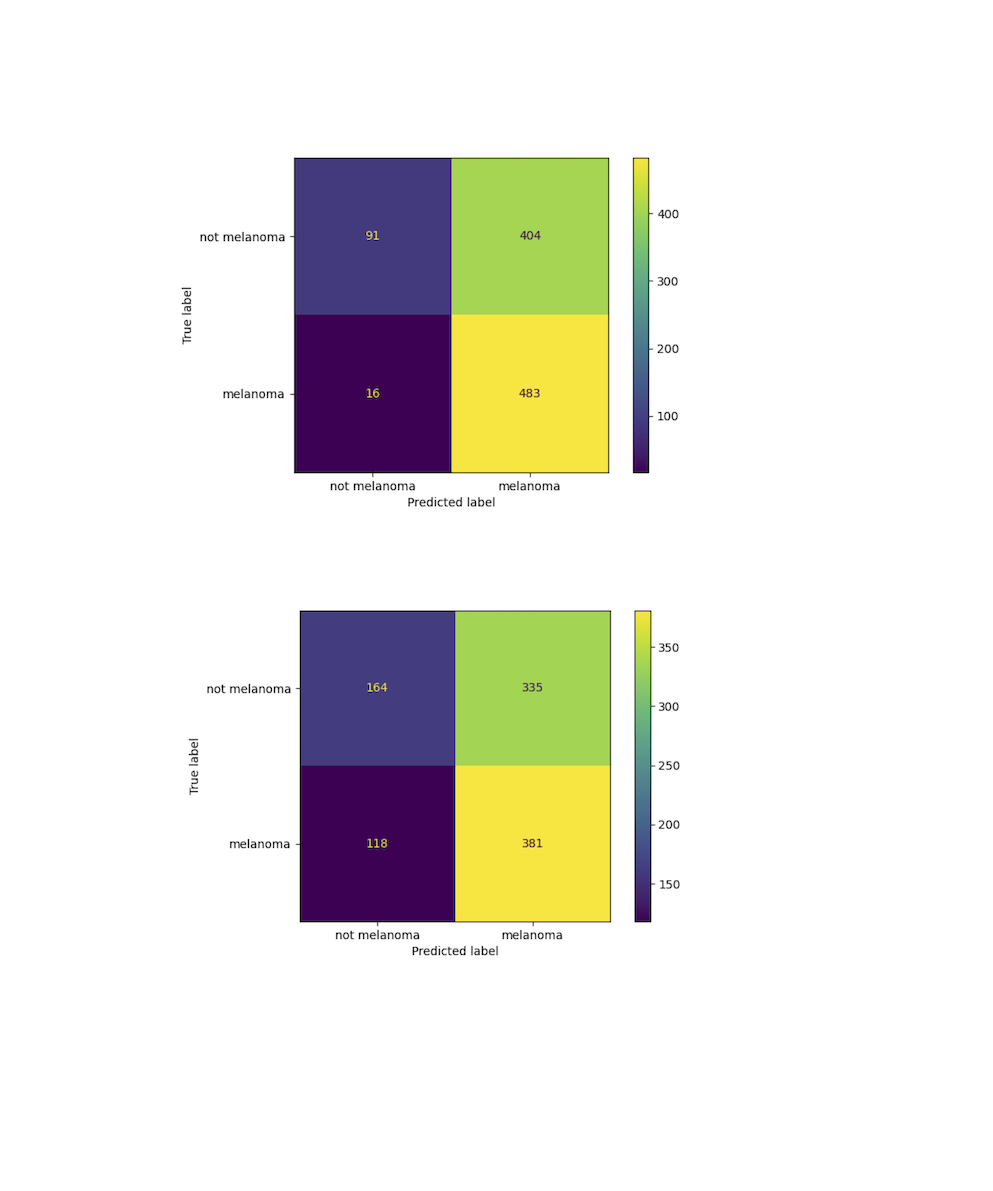

Supplement: Multimedia Appendix 1 [file derma-v8-e67551-s001.png]
